# Head and dental injuries or other dental problems in alpine sports

**DOI:** 10.1002/cre2.121

**Published:** 2018-07-27

**Authors:** Martina Schmid, Sabine Schädelin, Sebastian Kühl, Andreas Filippi

**Affiliations:** ^1^ Department of Oral Surgery, Oral Radiology and Oral Medicine and Centre of Dental Traumatology University of Basel Switzerland; ^2^ Clinical Trial Unit, Department of Clinical Research University Hospital Basel, University of Basel Switzerland

**Keywords:** alpine sports, dental trauma

## Abstract

In the hectic daily life, spending our leisure time outdoor in the mountains becomes more and more popular. Although information describing dental injuries in various sports is available, data specifically on dental trauma and other dental problems in alpine sports are lacking. Data of 1,128 alpinists were generated by using a standardized questionnaire. The questions focused on the frequency of head and dental injuries and other dental problems. The participating alpinists have been recruited through the network of alpine clubs and an outdoor equipment supplier.

Injuries were most frequently caused by a fall while doing alpine sports (14.7%, = 154). Of the participants, 8.2% (n = 93) suffered from a facial injury: 16% (n = 15) of these had a dental trauma. Other dental problems such as barodontalgia were more common than dental traumas. A sensation of pressure or pain was noticed in 13% (n = 135) of the participants at least once. Of all the participants who suffered pain, 54.1% reported one or several previous restorations. Pain medication was beneficial in 92.1%.

Dental traumas are rare while doing alpine sports but not negligible because of its requirement of lifelong therapy and incurs substantial treatment costs. An intraoral pain of a usually asymptomatic tissue occurred, in this sample of participants, nearly as often as in pilots and divers. Pain killer is an efficient tool for the alpinists to reduce the pain until they reach a lower altitude.

## 1 INTRODUCTION

Spending your free time outdoor in the mountains becomes, in the hectic daily life, more and more popular. Although information describing dental injuries in various sports is available, data specifically on dental trauma and other dental problems doing alpine sports are lacking.

Almost any sport can result in trauma, and the estimated global percentage of admissions for head injury related to sport are 10–12% (Jennett, 1996). The overall incidence of dental trauma in facial injuries is 48% (Gassner, Bösch, Tuli, & Emshoff, 1999) and in sports‐related facial trauma 50.1% (Gassner et al., 1999). Generally, articles on dental trauma due to sport report a frequency around 13–39% (Flanders & Bhat, 1995; Gutmann & Gutmann, 1995; Rodd & Chesham, 1997).

Various harms have not only been caused by a fall or rockfall but also by the changing ambient pressure provoking dental barotrauma.

Using a standardized questionnaire, 1,128 alpinists have been surveyed. A similar study design has often been used in comparable (published) sports‐related trauma articles (Fasciglione, Persic, Pohl, & Filippi, 2007; Gass, Kuhl, Connert, & Filippi, 2016; Hersberger, Krastl, Kuhl, & Filippi, 2012; Muller, Persic, Pohl, Krastl, & Filippi, 2008; Schildknecht, Krastl, Kuhl, & Filippi, 2012).

The aim of this study was to evaluate the occurrence of head and dental injuries such as dental problems while doing alpine sports.

## 2 MATERIAL AND METHODS

A total of 1,128 alpinists (defined as someone doing mountaineering sports above 1,500 mamsl) have been surveyed by using standardized questionnaire. The questionnaire contained 23 questions about the epidemiology (age, sex, and frequency of mountaineering), general facial and dental injuries, and other dental problems (Table 1). After getting the permission of Swissethics (Req‐2017‐00825), the questionnaire was uploaded to the provider Survey Monkey, San Mateo, CA, USA. The link to the created website (including the link to the questionnaire) was distributed through the network of alpine clubs and an equipment outdoor supplier.

**Table 1:** Questionnaire

1. What is your gender? (male/female)
2. How old are you? (under 18/18–25/26–45/46–65/over 65 years)
3. How long have you already been an alpinist? (since childhood/10 or more years/less than 10 years)
4. How often do you do alpine sports? (1× week/1× month/2–6× year/1× year/rarer on 1,500–2,500/2,500–3,500/3,500–4,500 or over 4,500 mamsl)
5. How willing are you to take a risk? (from 1 to 100)
6. Would you consider yourself as an experienced, average experienced, or not so experienced mountaineer?
7. Have you ever been hurt so badly that you had to see a doctor while being in great height? (yes/no)
8. If yes, what kind of injuries did you have? (bruise/scrape/muscle/ligament lesion/bone fracture/frostbite)
9. Have you ever been hurt in the face while being in great height? (yes/no)
10. If yes, what kind of face injuries? (laceration/scrape/bruise/dental trauma, other)
11. My “worst” trauma is caused by: (spill/rockfall/other)
12. Have you ever suffered from a feeling of pressure in the dental area or tooth ache while being in great height? (yes/no)
13. In which area was the pain located? (upper or lower jaw)
14. How long have you been (“usually”) in pain? (less than an hour/1 hr or more/one day or more)
15. How long have you been on the move before having the sensation of pressure/pain? (A couple of hours/a day/multiple days)
16. In what altitude did you have the sensation of pressure/tooth ache? (1,500–2,499/2,500–3,499/3,500–4,499/over 4,500 mamsl)
17. What treatment did you choose to eliminate the sensation of pressure/pain while being in the mountains? (nothing/pain killers/descending/cooling/other)
18. Did you experience a relief after the treatment? (yes/no/no, only short time the pain occurred very quickly again)
19. How would you describe the pain? (dull/sharp/throbbing/emissive)
20. Have you ever had pain in this tooth or in this area while not doing alpine sports? (Yes, long time ago./Yes, multiple times./Yes, shortly before the expedition/No, not that I could remember.)
21. Do you know if you have a filling, root filling, crown, or bridge? (No, not that I would know./Yes, I have a filling/root filling/crown/bridge/other)
22. Do you know if you press your teeth during night? (no/yes, my partner has already told me. But I have not recognized so far./Yes, I have a feeling of tension in the area of jaw.)
23. Do you have a bite splint? (yes/no)

The statistical evaluation assessed the frequency of alpine sports, the occurrence of injuries, and dental problems. Patient characteristics, self‐reported medical history, and routine as alpinists are presented descriptively. Categorical data are presented as frequencies and percentages. For continuous variables, the mean, the standard deviation, or the median and the interquartile range are presented as appropriate. The proportion of alpinists with pain or pressure is estimated together with its 95% confidence interval (CI) according to Blaker (2000). The level of significance was determined to be *P* ≤ 0.05.

## 3 RESULTS

More males (64.2%) than females (32.8%) participated in this study (Table 2). Most of the mountaineers have been between 26 and 65 years (80.4%). Four hundred eighty‐four people (42.9%) reported that their experience in mountaineering goes back to their childhood. Three hundred sixty‐three (32.2%) claimed to have more than 10 years and only 245 (21.7%) less than 10 years of mountaineering experience (Table 2).

**Table 2:** Participant characteristics

V1	Overall
*n*	1,128
Sex (%)	
Male	724 (64.2)
Female	370 (32.8)
No response	34 (3.0)
Age (%)	
<18	8 (0.7)
18–25	115 (10.2)
26–45	490 (43.4)
46–65	417 (37.0)
>65	62 (5.5)
No response	36 (3.2)
Experience (%)	
Less than 10 years	245 (21.7)
More than 10 years	363 (32.2)
Since childhood	484 (42.9)
No response	36 (3.2)
Self.assessment (%)	
Little experience	372 (33.0)
Average experience	549 (48.7)
Much experience	173 (15.3)
No response	34 (3.0)

The sample includes experienced participants who often reach high altitudes up to participants who rarely ascend to great altitudes. Two hundred sixty alpinists (23%) are doing sports once a week between 1,500 and 2,500 mamsl, 363 mountaineers (32.2%) are twice up to six times a year between 2,500 and 3,500 mamsl (Table 3). Two hundred fourteen participants (19%) are twice up to six times a year around 3,500–4,500 mamsl, and six alpinists (0.5%) are even weekly over 4,500 mamsl.

**Table 3:** Participants routine

*N* (%)	Once a week	Once a month	Twice to six times	Once a year	Less than once a year
Less than 1,500 mamsl/no response	788 (69.9)	649 (57.5)	321 (28.5)	678 (60.1)	774 (68.6)
1,500–2,500 mamsl	260 (23.0)	209 (18.5)	198 (17.6)	23 (2.0)	19 (1.7)
2,500–3,500 mamsl	64 (5.7)	231 (20.5)	363 (32.2)	138 (12.2)	22 (2.0)
3,500–4,500 mamsl	10 (0.9)	36 (3.2)	214 (19.0)	206 (18.3)	67 (5.9)
>4500 mamsl	6 (0.5)	3 (0.3)	32 (2.8)	83 (7.4)	246 (21.8)

### 3.1 Injuries

Most alpinists (*n* = 91, 59%) had suffered from a muscle, tendon, or ligament injury. Sixty‐one mountaineers (40%) had a bone fracture(s) while being in the mountains (Table 4). Sixteen percent of the participants who reported to have ever suffered from a facial injury (*n* = 93) had a dental trauma. In most of the facial‐injured mountaineers, 61.3% had a graze or laceration (45%; Table 5).

**Table 4:** Injuries

		Yes		No	
		*n*	%	*n*	%
	All	154	100	894	100
Contusion	Contusion	67	44	0	0
	No	87	56	894	100
Graze	Graze	54	35	0	0
	No	100	65	894	100
Muscle, tendon, or ligament injury	Muscle, tendon, or ligament injury	91	59	0	0
	No	63	41	894	100
Bone fracture	Bone fracture	61	40	0	0
	No	93	60	894	100
Frostbite	Frostbite	24	16	0	0
	No	130	84	894	100

**Table 5:** Facial injuries

		Yes		No		Rockfall		Fall	
		*n*	%	*n*	%	*n*	%	*n*	%
	All	93	100.0	951	100.0	14	100.0	71	100.0
Laceration	Laceration	42	45.2	0	0.0	10	71.4	32	45.1
	No	51	54.8	951	100.0	4	28.6	39	54.9
Graze	Graze	57	61.3	0	0.0	8	57.1	49	69.0
	No	36	38.7	951	100.0	6	42.9	22	31.0
Contusion	Contusion	33	35.5	0	0.0	6	42.9	27	38.0
	No	60	64.5	951	100.0	8	57.1	44	62.0
Dental trauma	Dental trauma	15	16.1	0	0.0	4	28.6	10	14.1
	No	78	83.9	951	100.0	10	71.4	61	85.9
Cerebral concussion	Cerebral concussion	4	4.3	2	0.2	1	7.1	3	4.2
	No	89	95.7	949	99.8	13	92.9	68	95.8
Bone fracture	Bone fracture	6	6.5	0	0.0	0	0.0	6	8.5
	No	87	93.5	951	100.0	14	100.0	65	91.5
Frostbite	Frostbite	8	8.6	0	0.0	0	0.0	4	5.6
	No	85	91.4	951	100.0	14	100.0	67	94.4

### 3.2 Pressure and pain

A total of 1036 participants answered the questions regarding pressure or pain. Ninety‐two did not answer this question. In total, 135 (or 13%; CI [11.1, 15.2]) participants reported a sensation of pressure or pain at least once. Eight alpinists reported repeated pain or pressure. Forty‐four alpinists (32.6%) suffered 1 hr or longer, 27 (20%) a day or longer, and 53 (39.3%) had less than an hour toothache. The location of the pain was in most of the participants the upper jaw. The mountaineers had been mostly a few hours “climbing/hiking” before their tooth/teeth started hurting (Table 6). Of the participants, 35.6% reported pain at 2,500–4,955 mamsl (Table 7).

**Table 6:** Pain location, time duration, and time to pain in participants who reported pain

	Overall
*n*	135
Pain duration %	
1 hr or longer	44 (32.6)
1 day or longer	27 (20.0)
Less than an hour	53 (39.3)
No response	11 (8.1)
Pain region (%)	
Upper jaw	87 (64.4)
Lower jaw	36 (26.7)
No response	12 (8.9)
Time to pain (%)	
A day	36 (26.7)
Multiple days	19 (14.1)
Some hours	69 (51.1)
No response	11 (8.1)

**Table 7:** Altitude, participants reported pain (including only participants who reported to have had pain)

	All	
Pain altitude	*n*	%
1,500–2,499 mamsl	37	27.4
2,500–3,499 mamsl	48	35.6
3,500–4,499 mamsl	28	20.7
Over 4,500 mamsl	11	8.1

### 3.3 Response to pain

Thirty‐nine out of 135 alpinists took pain medication with a 92.3% success rate (long or short term). Ten out of 135 mountaineers had, due to the pain, to descend—with 70% long‐term success of being pain free at a lower altitude (Table 8). Most of the alpinists (*n* = 48) described the pain to be dull, second most they reported that they felt it throbbing (Table 9).

**Table 8:** Success of treatment

	*N*	Success (%)	No success (%)	Short‐term success (%)
Do nothing	82	31 (37.8)	40 (48.8)	2 (2.4)
Pain medication	39	26 (66.7)	3 (7.7)	10 (25.6)
Descend	10	7 (70.0)	0 (0.0)	3 (30.0)
Cooling	4	3 (75.0)	0 (0.0)	1 (25.0)

**Table 9:** Description of pain

	All	
Pain quality	*n*	%
Emissive	14	10.4
Throbbing	20	14.8
Throbbing emissive	4	3.0
Sharp	11	8.1
Sharp throbbing	7	5.2
Dull	48	35.6
Dull emissive	5	3.7
Dull throbbing	9	6.7
Dull throbbing emissive	1	0.7
Dull stabbing	3	2.2
Dull stabbing emissive	1	0.7

Of the participants, 45.2% could not remember if they ever had a treatment on that specific tooth. Sixty‐three alpinists knew they had at least once or even repeated a treatment (Table 10).

**Table 10:** Previous pain

Previous pain	*N*	%
Yes, long time ago.	33	24.4
Yes, repeatedly.	30	22.2
No, not that I could remember.	61	45.2
No response	11	8.1

**Figure 1:**
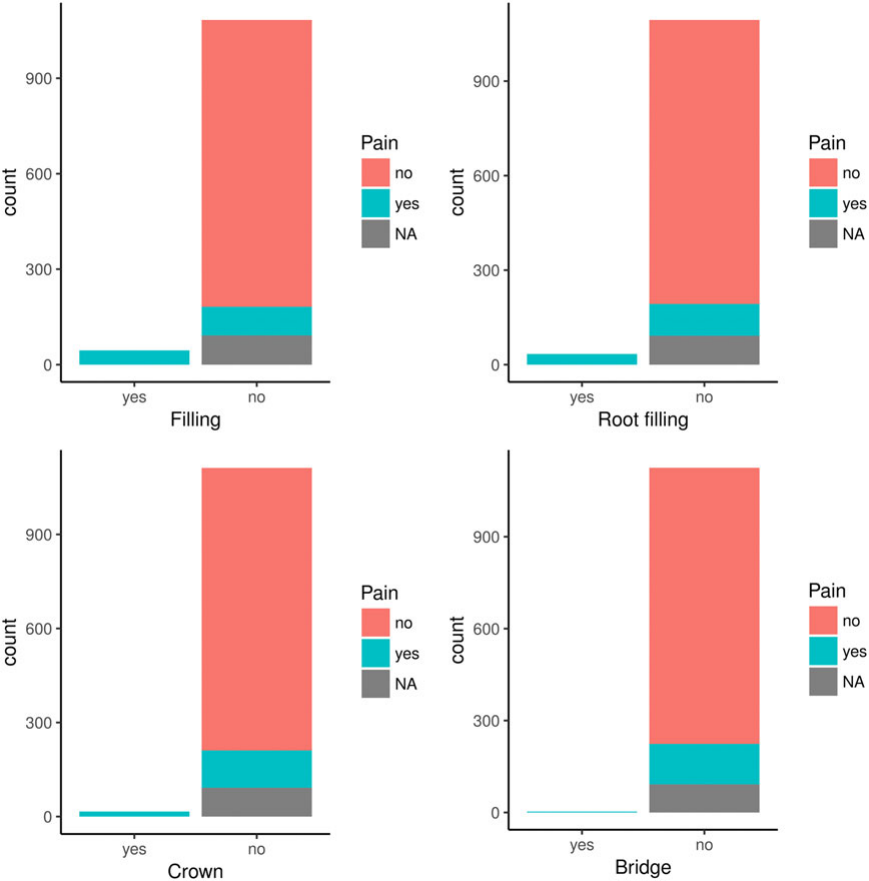
Pain by previous treatment

There were 73 participants who reported to have had a previous treatment(s) (filling, root canal treatment, crown, and bridge) on the aching tooth. In total, 54.1% (95% CI [45.5, 62.6]) of all participants who suffered pain also reported one or several previous treatments (Figure 1).

### 3.4 Bruxism

Of the participants, 11.9% (*n* = 45) reported pain at least once up in the mountains and do grind or press their teeth. Three hundred seventy‐eight of the participants have been told to be grinding/pressing their teeth by their partner or have a sensation of tension in the area of their jaw. Of the climbers, 11.9% have a bite splint (Table 11).

**Table 11:** Bruxism by pain

		No		Yes		All	
		*n*	%	*n*	%	*n*	%
Bruxism	Yes, I have a feeling of tension in the area of the jaw.	126	14.0	24	17.8	150	13.3
	Yes, my partner has already told me. But I have not recognized so far.	207	23.0	21	15.6	228	20.2
	No	563	62.5	82	60.7	645	57.2
	No response	5	0.6	8	5.9	105	9.3
Bite splint	Yes	100	11.1	16	11.9	116	10.3
	No	794	88.1	105	77.8	899	79.7
	No response	7	0.8	14	10.4	113	10.0

## 4 DISCUSSION

This study evaluated the frequency of head and dental injuries such as dental problems while doing alpine sports. Of all participants, 14.7% (*n* = 154) had injuries with a need of medical assistance by a doctor. Most alpinists, 91 out of 154 (59%), suffered from either muscle, tendon, or ligament injury while being in the mountains. In comparison, “open wounds” are the most often injury in ice climbing (52%; Schoffl, Morrison, Schoffl, & Kupper, 2012). Most of the injuries of the mountaineers (46.1%, *n* = 71) have been caused by a fall as also surveyed in alpine skiing (42%; Gassner, Vàsquez Garcia, Leja, & Stainer, 2000) or mountaineering/rock climbing (Addiss & Baker, 1989; Bowie, Hunt, & Allen Jr, 1988).

There is an association between facial fractures and dental injuries (Lieger, Zix, Kruse, & Iizuka, 2009). Sixteen percent of the participants who reported to have ever suffered from a facial injury had a dental trauma, rather rarely in comparison with 48.25% of the overall incidence of dental trauma in facial injuries and in sports‐related facial trauma (Gassner et al., 2000). Generally, articles on dental trauma due to sport have incidence rates around 13–39% (Flanders & Bhat, 1995; Gutmann & Gutmann, 1995; Rodd & Chesham, 1997). Up to 35% of children and adolescents suffer from accidents involving permanent teeth (Ivancic Jokic, Bakarcic, Fugosic, Majstorovic, & Skrinjaric, 2009). Owing to their exposed position (Altun et al., 2009), the upper front teeth and the upper jaw are most often affected. Even if severe dental traumas are not as common as in contact sports, it is still important because of its requirement of therapy and treatment costs (Wong & Kolokotsa, 2004).

Thirteen percent of the participants reported a sensation of pressure or toothache only while being up in the mountains. An intraoral pain of a usually asymptomatic tissue caused by barometric changes (usually ascent or descent) is called barodontalgia (Zadik, 2009a; Zadik & Drucker, 2011). The prevalence of barodontalgia is until now only examined in pilots/air travelers (11%) and divers (10.2%; Zadik, 2010; Zanotta, Dagassan‐Berndt, Nussberger, Waltimo, & Filippi, 2014).

This discomfort can be produced by odontogenic or non‐odontogenic origin (Zadik, 2009a; Zadik, 2009b). The pathophysiology is until now not fully investigated (Zadik & Drucker, 2011). There are several cofactors leading to the pain such as odontogenic inflammation, sinusitis, secondary caries, necrotic pulp, apical periodontitis, insufficient restorations up to the dentin, or a recently performed treatment by a dentist (Carlson, Halverson, & Triplett, 1983; Kieser & Holborow, 1997; Lurie et al., 2007; Robichaud & McNally, 2005; von See, Rücker, & Gellrich, 2010; Zadik, 2006; Zadik, 2009a). In most cases, barodontalgia is an exacerbation of a pre‐existing subclinical intraoral disease (Donovan et al., 2008). Of all participants who suffered from pain at a specific height, 54.1% also reported one or several previous dental treatments. Most likely, there is a recall bias and/or maybe the climbers remember “better” if they have a filling/crown if they ever had severe pain.

As the data of this study are collected through questionnaires, there cannot be made any statement of the oral pathology or reconstruction and its condition. Existing oral diseases or insufficient fillings/crowns are maybe responsible for the barodontalgia found in this study. For a layperson, it is difficult to differentiate between tooth and composite filling, and therefore, it might be a source of error.

Scuba divers experienced the pain more frequently while there is an increase of pressure (Lurie et al., 2007; von See et al., 2010; Zadik, 2009a). In this study, most of the mountaineers had been a few hours up in the mountains before they had pain, and 35.6% reported it between 2,500 and 3,499 mamsl. There is no aggravation if the tooth is longer exposed to a lower barometric pressure. The symptoms are rarely that severe that the scuba diver, submariners, pilots, and airline passenger might do pain‐induced mistakes and, as a consequence, pose themselves to a serious safety risk (Zadik, 2006; Zadik & Drucker, 2011). As the prevalence of barodontalgia from divers, submariners, pilots, and airline passengers is comparable with the alpinists, it can be concluded that it is a risk for the alpinists as well. Of the alpinists, 7.4% (*n* = 10) reported that they had to descend due to the pain—with 70% long‐term success of being pain free at a lower altitude. Thirty percent of the mountaineers took pain medication with 92.3% success rate; 25.6% benefited only short term, but it might be enough to find a shelter or get out of a danger area. Pain killer is a great tool for the alpinists to reduce the pain until they reach a lower altitude.

Most of the participating alpinists reported to have the pain located in the upper jaw (64.4%), where a similarity to scuba divers (Zanotta et al., 2014) is found. Unlike those two studies, pilots located the pain equally in the upper and lower jaw (Jagger, Jackson, & Jagger, 1997).

Because severe dental traumas are very rare (*n* = 15) in mountaineering, there is no exorbitant need in promoting bite splint. Treatment recommendations for barodontalgia are the same as for scuba divers (Zanotta et al., 2014). If the patient suffered from a barodontalgia, the dentist should not only concentrate on anamneses and clinical examination (Zadik, 2009a). He/she should especially focus on recently performed treatments, pre‐existing symptoms (such as secondary caries), point of time when the pain developed, and the quality of pain (Zadik, 2009a). After a conservative dental treatment under local anesthesia, it should be recommended not to go up to a “very high altitude” for the upcoming 24 hr (Zadik & Drucker, 2011); after oral surgery 7 days (Robichaud & McNally, 2005).

Annual check‐ups or before a planned longer stay at a high altitude should be done. Further investigation of dental injuries in alpine sports would be valuable in order to make guidelines regarding the use of mouthguards/bite splints and the treatment of barodontalgia.

## CONFLICT OF INTEREST

The authors declare that they have no conflict of interests.

## References

[cre2121-bib-0001] Addiss, D. G. , & Baker, S. P. (1989). Mountaineering and rock‐climbing injuries in us national parks. Annals of Emergency Medicine, 18, 975–979. 2764331 10.1016/s0196-0644(89)80463-9

[cre2121-bib-0002] Altun, C. , Ozen, B. , Esenlik, E. , Guven, G. , Gürbüz, T. , Acikel, C. , … Akbulut, E. (2009). Traumatic injuries to permanent teeth in Turkish children, Ankara. Dental Traumatology, 25, 309–313. 19583580 10.1111/j.1600-9657.2009.00778.x

[cre2121-bib-0003] Blaker, H. (2000). Confidence curves and improved exact confidence intervals for discrete distributions. The Canadian Journal of Statistics, 28, 783–798.

[cre2121-bib-0004] Bowie, W. S. , Hunt, T. K. , & Allen, H. A. Jr. (1988). Rock‐climbing injuries in Yosemite National Park. The Western Journal of Medicine, 149, 172–177. 3247732 PMC1026367

[cre2121-bib-0005] Carlson, O. G. , Halverson, B. A. , & Triplett, R. G. (1983). Dentin permeability under hyperbaric conditions as a possible cause of barodontalgia. Undersea Biomedical Research, 10, 23–28. 6868178

[cre2121-bib-0006] Donovan, T. E. , Becker, W. , Brodine, A. H. , Burgess, J. O. , Cagna, D. R. , & Summit, J. B. (2008). Annual review of selected dental literature: report of the Committee on Scientific Investigation of the American Academy of Restorative Dentistry. The Journal of Prosthetic Dentistry, 100, 110–141. 18672128 10.1016/S0022-3913(08)60159-6

[cre2121-bib-0007] Fasciglione, D. , Persic, R. , Pohl, Y. , & Filippi, A. (2007). Dental injuries in inline skating—Level of information and prevention. Dental Traumatology, 23, 143–148. 17511835 10.1111/j.1600-9657.2005.00415.x

[cre2121-bib-0008] Flanders, R. A. , & Bhat, M. (1995). The incidence of orofacial injuries in sports: A pilot study in Illinois. Journal of the American Dental Association (1939), 126, 491–496. 7722111 10.14219/jada.archive.1995.0213

[cre2121-bib-0009] Gass, M. , Kuhl, S. , Connert, T. , & Filippi, A. (2016). Dental trauma in showjumping—A trinational study between Switzerland, France and Germany. Dental Traumatology, 32, 174–179. 26542314 10.1111/edt.12242

[cre2121-bib-0010] Gassner, R. , Bösch, R. , Tuli, T. , & Emshoff, R. (1999). Prevalence of dental trauma in 6000 patients with facial injuries. Oral Surgery, Oral Medicine, Oral Pathology, Oral Radiology, and Endodontics, 87, 27–33. 10.1016/s1079-2104(99)70290-8 9927076

[cre2121-bib-0011] Gassner, R. , Vàsquez Garcia, J. , Leja, W. , & Stainer, M. (2000). Traumatic dental injuries and alpine skiing. Endodontics & Dental Traumatology, 16, 122–127. 11202868 10.1034/j.1600-9657.2000.016003122.x

[cre2121-bib-0012] Gutmann, J. L. , & Gutmann, M. S. (1995). Cause, incidence, and prevention of trauma to teeth. Dental Clinics of North America, 39, 1–13. 7890099

[cre2121-bib-0013] Hersberger, S. , Krastl, G. , Kuhl, S. , & Filippi, A. (2012). Dental injuries in water polo, a survey of players in Switzerland. Dental Traumatology, 28, 287–290. 22107072 10.1111/j.1600-9657.2011.01083.x

[cre2121-bib-0014] Ivancic Jokic, N. , Bakarcic, D. , Fugosic, V. , Majstorovic, M. , & Skrinjaric, I. (2009). Dental trauma in children and young adults visiting a University Dental Clinic. Dental Traumatology, 25, 84–87. 19208016 10.1111/j.1600-9657.2008.00711.x

[cre2121-bib-0015] Jagger, R. G. , Jackson, S. J. , & Jagger, D. C. (1997). In at the deep end—An insight into scuba diving and related dental problems for the GDP. British Dental Journal, 183, 380–382. 9419946 10.1038/sj.bdj.4809515

[cre2121-bib-0016] Jennett, B. (1996). Epidemiology of head injury. Journal of Neurology, Neurosurgery, and Psychiatry, 60, 362–369. 10.1136/jnnp.60.4.362 PMC1073884 8774396

[cre2121-bib-0017] Kieser, J. , & Holborow, D. (1997). The prevention and management of oral barotrauma. The New Zealand Dental Journal, 93, 114–116. 9470443

[cre2121-bib-0018] Lieger, O. , Zix, J. , Kruse, A. , & Iizuka, T. (2009). Dental injuries in association with facial fractures. Journal of Oral and Maxillofacial Surgery, 67, 1680–1684. 19615582 10.1016/j.joms.2009.03.052

[cre2121-bib-0019] Lurie, O. , Zadik, Y. , Einy, S. , Tarrasch, R. , Raviv, G. , & Goldstein, L. (2007). Bruxism in military pilots and non‐pilots: tooth wear and psychological stress. Aviation, Space, and Environmental Medicine, 78, 137–139. 17310886

[cre2121-bib-0020] Muller, K. E. , Persic, R. , Pohl, Y. , Krastl, G. , & Filippi, A. (2008). Dental injuries in mountain biking‐ a survey in Switzerland, Austria, Germany and Italy. Dental Traumatology, 24, 522–527. 18821955 10.1111/j.1600-9657.2008.00660.x

[cre2121-bib-0021] Robichaud, R. , & McNally, M. E. (2005). Barodontalgia as a differential diagnosis: symptoms and findings. Journal of the Canadian Dental Association, 71, 39–42. 15649340

[cre2121-bib-0022] Rodd, H. D. , & Chesham, D. J. (1997). Sports‐related oral injury and mouthguard use among Sheffield school children. Community Dental Health, 14, 25–30. 9114546

[cre2121-bib-0023] Schildknecht, S. , Krastl, G. , Kuhl, S. , & Filippi, A. (2012). Dental injury and its prevention in Swiss rugby. Dental Traumatology, 28, 465–469. 22300191 10.1111/j.1600-9657.2012.01115.x

[cre2121-bib-0024] Schoffl, V. , Morrison, A. , Schoffl, I. , & Kupper, T. (2012). The epidemiology of injury in mountaineering, rock and ice climbing. Medicine and Sport Science, 58, 17–43. 22824837 10.1159/000338575

[cre2121-bib-0025] von See, C. , Rücker, M. , & Gellrich, N. C. (2010). Barodontalgia etiology, diagnosis and treatment considerations. Endodontie, 19, 43–48.

[cre2121-bib-0026] Wong, F. S. , & Kolokotsa, K. (2004). The cost of treating children and adolescents with injuries to their permanent incisors at a dental hospital in the United Kingdom. Dental Traumatology, 20, 327–333. 15522054 10.1111/j.1600-9657.2004.00263.x

[cre2121-bib-0027] Zadik, Y. (2006). Barodontalgia due to odontogenic inflammation in the jawbone. Aviation, Space, and Environmental Medicine, 77, 864–866. 16909883

[cre2121-bib-0028] Zadik, Y. (2009a). Barodontalgia. Journal of Endodontia, 35, 481–485. 10.1016/j.joen.2008.12.004 19345791

[cre2121-bib-0029] Zadik, Y. (2009b). Aviation dentistry: Current concepts and practice. British Dental Journal, 206, 11–16. 19132029 10.1038/sj.bdj.2008.1121

[cre2121-bib-0030] Zadik, Y. (2010). Barodontalgia: What have we learned in the past decade? Oral Surgery, Oral Medicine, Oral Pathology, Oral Radiology, and Endodontics, 109, E65–E69. 10.1016/j.tripleo.2009.12.001 20303049

[cre2121-bib-0031] Zadik, Y. , & Drucker, S. (2011). Diving dentistry: A review of the dental implications of scuba diving. Australian Dental Journal, 56, 265–271. 21884141 10.1111/j.1834-7819.2011.01340.x

[cre2121-bib-0032] Zanotta, C. , Dagassan‐Berndt, D. , Nussberger, P. , Waltimo, T. , & Filippi, A. (2014). Barodontalgias, dental and orofacial barotraumas: A survey in Swiss divers and caisson workers. Swiss Dental Journal, 124, 510–519. 24853026 10.61872/sdj-2014-05-01

